# Evaluation of myeloid metaplasia in canine mixed mammary tumors

**DOI:** 10.1186/1753-6561-7-S2-P13

**Published:** 2013-04-04

**Authors:** Cecilia B Campos, Patricia A Auler, Angelica C Bertagnolli, Enio Ferreira, Gustavo M Ribeiro, Ana PM Dias, Liliane C Campos, Geovanni D Cassali

**Affiliations:** 1 Department of General Pathology, Federal University of Minas Gerais (UFMG), Belo Horizonte, Brazil; 2 Department of Medical Sciences, Federal University of Ouro Preto (UFOP), Ouro Preto, Brazil

## Background

Mixed tumors are among the most frequent mammary neoplasms in female dogs. Some of these tumors present bone marrow associated with the newly formed osseous tissue, characteristic of myeloid metaplasia. Our aims were to evaluate the occurrence of these lesions in a series of mixed tumors, and to determine tumor histomorphological characteristics.

## Materials and methods

384 mammary mixed tumors from 289 animals have been reviewed. Lesions were classified according to the presence of osseous metaplasia associated with myeloid metaplasia or extramedullary hematopoiesis. Myeloid metaplasia characterization was determined from the morphological characteristics and organization of cells and adjacent tissues.

## Results

The 384 cases included 206 benign and 178 carcinomas in mixed tumors. Osseous metaplasia was present in 16.1% and calcified areas exclusively in 3.1% of lesions. Among all osseous metaplasia, 33.9% presented some type of extramedullary hematopoiesis, of which 71.4% were classified as myeloid metaplasia and 28.6% as extramedullary hematopoiesis. Myeloid metaplasia cases consisted of 67% benign mixed tumors and 33% carcinomas in mixed tumors. Myeloid metaplasia was observed in 24% of mixed tumors containing osseous metaplasia and in 4% of all mixed tumors analyzed.

## Conclusions

Despite these results and considering the low frequency of this lesion, additional studies are needed to understand the implications of myeloid metaplasia in canine mammary mixed tumors.

## Financial support

FAPEMIG, CAPES and CNPq.

